# Safety and efficacy of SGLT2 inhibitors in heart failure patients with ischemic and non-ischemic etiologies: a systematic review and meta-analyses

**DOI:** 10.1186/s43044-025-00623-5

**Published:** 2025-03-19

**Authors:** Hasan Fareed Siddiqui, Adam Bilal Khan, Muhammad Moiz Nasir, Taleen Hashmi, Aisha Fareed Siddiqui, Hanzla Asim, Bushra Iqtidar Siddiqui

**Affiliations:** 1 Department of Internal Medicine, Dow University of Health Sciences, Baba-e-Urdu Road, Karachi, 74200 Pakistan; 2 Department of Internal Medicine, Aga Khan University Hospital, Karachi, Pakistan

**Keywords:** Sodium-glucose cotransporter 2 inhibitors, Heart failure, Cardiovascular death, Hospitalization due to heart failure, All-cause mortality, Meta-analyses

## Abstract

**Background:**

Sodium-glucose cotransporter 2 inhibitors (SGLT2is) show promise as a therapy for heart failure (HF); however, the safety and efficacy of SGLT2i in different HF etiologies are uncertain, thus arising the need for a meta-analyses.

**Main text:**

PubMed and Scopus were queried until May 2023 for studies comparing SGLT2i with placebo in HF patients with ischemic and non-ischemic etiologies. Meta-analyses were performed using risk ratio and hazard ratio. A fixed effect model was used. Outcomes assessed were hospitalization due to HF (HHF), cardiovascular death (CVD), CVD/HHF, all-cause mortality, volume depletion, fracture, and discontinuation of drug due to adverse effects. Four RCTs were included (15,676 patients). Analysis revealed no significant differences in CVD/HHF between ischemic [HR: 0.77 (0.70–0.86) *P* < 0.00001] and non-ischemic patients [HR: 0.72 (0.65–0.80) *P* < 0.00001] using SGLT2i (*P* = 0.35). Significant reductions were seen in HHF in both ischemic [RR 0.74 (0.65–0.84) *P* < 0.00001] and non-ischemic [RR 0.68 (0.59–0.78) *P* < 0.00001] patients (*P* = 0.39), with the effect more notable in the non-ischemic cohort. However, CVD significantly decreased in non-ischemic patients [RR 0.78 (0.63–0.95) *P* = 0.01], whereas no significant reduction was noted in ischemic patients [RR 0.94 (0.80–1.10) *P* = 0.43] (*P*-interaction = 0.15). All-cause mortality was significantly reduced in non-ischemic patients [RR 0.80 (0.67–0.96) *P* = 0.02] but not in ischemic patients [RR 0.96 (0.83–1.10) *P* = 0.52]. No significant safety events were observed in the SGLT2i cohort including volume depletion [RR 1.08 (0.94–1.25) *P* = 0.26], fracture [RR 1.02 (0.77–1.36) *P* = 0.88], or discontinuation of drug due to adverse effects [RR 0.97 (0.86–1.10) *P* = 0.65].

**Conclusion:**

Similar CVD/HHF outcomes for ischemic and non-ischemic patients with SGLT2i. Significant HHF reductions in both groups. Non-ischemic patients showed greater improvements in CVD and all-cause mortality. However, no subgroup difference between ischemic and non-ischemic cause of heart failure was noted in our analysis.

## Background

With an impact spanning across approximately 64.3 million patients globally, heart failure (HF) has emerged as a prevailing and escalating public health concern in recent times. In the USA, the statistics are concerning, with a notable 30-day readmission rate for all-cause mortality standing at 19% [1–3]. In line with this evolving landscape, the 2021 guidelines of the European Society of Cardiology (ESC) recommend the utilization of sodium-glucose transport 2 inhibitors (SGLT2i) in the management of stable and chronic HF with reduced ejection fraction (HFrEF) [4]. SGLT2i, initially grouped within the realm of traditional antihyperglycemic agents, has now firmly established themselves as a cornerstone treatment modality for HF.

Remarkably, the EMPA-REG OUTCOME trial (Empagliflozin Cardiovascular Outcome Event Trial in Type 2 Diabetes Mellitus Patients—Removing Excess Glucose; NCT01131676) disclosed a striking 35% reduction in hospitalization due to heart failure (HHF) attributed to the implementation of empagliflozin. This phenomenon was subsequently reaffirmed in cardiovascular outcome trials that explored the effects of canagliflozin and dapagliflozin, underlining the consistent and robust nature of this benefit [5–7]. The compelling insights from these trials have propelled SGLT2i into the forefront of potential therapeutic approaches for managing heart failure, sparking heightened interest, and optimism within the medical community.

Nevertheless, it is important to highlight that the exploration of SGLT2i's safety and efficacy in patients with heart failure, distinguished by their underlying etiologies (ischemic vs. non-ischemic), remains a relatively understudied area. The traditional classification of heart failure into either ischemic or non-ischemic causes oversimplifies a complex reality. When multiple health conditions coexist, pinpointing the exact origin becomes challenging. Currently, no widely accepted definition universally defines the underlying cause of heart failure. [8] Furthermore, given the discrepancies and incongruities present in existing literature, it becomes pivotal to delve into the ramifications associated with the etiology of heart failure. Initially, heart failure with reduced ejection fraction (HFrEF) originating from ischemic causes could potentially herald a more unfavorable prognosis in contrast to HFrEF attributed to non-ischemic factors [9]. Additionally, the influence of specific treatments for HFrEF, such as the implantable cardioverter-defibrillator, might be influenced by the underlying cause [10], hinting at potential variations in treatment response based on the particular etiology. Consequently, we conducted a systematic review and meta-analysis to comprehensively assess the safety and effectiveness of SGLT2i in the context of ischemic versus non-ischemic heart failure.

## Methods

### Data source and search strategy

This systematic review adhered to the guidelines outlined by the Preferred Reporting Items for Systematic Reviews and Meta-Analyses (PRISMA) [11]. Relevant studies were electronically searched using databases such as MEDLINE (via PubMed), Scopus, Cochrane CENTRAL, and EMBASE from inception up to May 2023 using the search string (("Sodium-Glucose Transporter 2 Inhibitors"[Mesh] OR "canagliflozin" OR "dapagliflozin" OR "empagliflozin" OR "ertugliflozin" OR "ipragliflozin" OR "luseogliflozin" OR "sotagliflozin") AND ("Heart Failure"[Mesh] OR "heart failure" OR "HF") AND ("Ischemia"[Mesh] OR "ischaemic" OR "Non-Ischemic"[Mesh] OR "non-ischaemic" OR "ischemic" OR "Nonischemic" OR "Non-Ischemic Cardiomyopathy" OR "NICM" OR "aetiology")).

### Study selection and eligibility criteria

A comprehensive evaluation encompassed a range of editorials, letters, and meta-analyses, encompassing both published and unpublished works, to identify potential eligible studies. Only studies written in the English language were deemed suitable for inclusion. For effective reference management, duplicates were identified and removed using EndNote reference management software (version 20.2.1, Clarivate Analytics). The remaining articles underwent a dual-stage review process conducted by two independent reviewers (A.B.K and M.M.N.). This process involved an initial assessment of titles and abstracts, followed by a comprehensive evaluation of full-text content. Any disparities in article selection were resolved through deliberation, and if necessary, a third investigator (H.F.S) was consulted to reach a consensus.

The central aim of these meta-analyses was to assess the effects of SGLT2 inhibitors (SGLT2is) on patients with heart failure, with a specific emphasis on analyzing the data by underlying causes (ischemic versus non-ischemic). Inclusion criteria for studies encompassed: (1) a comparison between an SGLT2i group and a placebo group, (2) the inclusion of heart failure patients, (3) reporting of primary outcomes of interest, (4) availability of data stratified by heart failure etiology (ischemic vs. non-ischemic), and (5) trial size exceeding 1000 participants. The primary outcomes examined were the composite of cardiovascular death and hospitalization due to heart failure (CVD/HHF), along with CV death, hospitalization due to heart failure (HHF), and all-cause death. Secondary outcomes encompassed discontinuation of drugs due to adverse events, hypoglycemic events, and amputation events.

### Data extraction and quality assessment

For each if the selected randomized controlled trial (RCT), their baseline patient characteristic, demographics, outcomes, and safety events were extracted. Risk of bias was assessed using the Cochrane risk of bias tool for randomized trials (RoB 2) [12]. Both the data extraction and quality assessment were performed by two reviewers (M.M.N and A.B.K). Any discrepancies were resolved through discussion with a third reviewer (H.F.S).

### Statistical analysis

Review Manager (version 5.3; Cochrane collaboration) was used to analyze the pooled data statistically [13]. The dichotomous data extracted from the studies comparing SGLT2i with placebo in ischemic and non-ischemic patients was analyzed by calculating a pooled risk ratio (RR) and their 95% confidence intervals, using the Mantel–Haenszel method for all outcomes except the composite of CV death and HHF; Hazards ratio and their 95% confidence intervals were pooled using the generic-inverse variance method for this outcome. To evaluate heterogeneity, we employed the Higgins *I*^2^ statistic, wherein values less than 50% indicated minimal heterogeneity, while values exceeding 50% signified substantial heterogeneity [11]. Furthermore, for outcomes demonstrating heterogeneity beyond 50%, a sensitivity analysis using a leave-one-out approach was conducted to explore potential underlying factors. Visual representation of the analysis was achieved through the creation of forest plots. Additionally, for each outcome, funnel plots were generated and visually inspected to gauge the presence of potential publication bias.

## Results

### Search results and patient characteristics

**Fig. 1:**
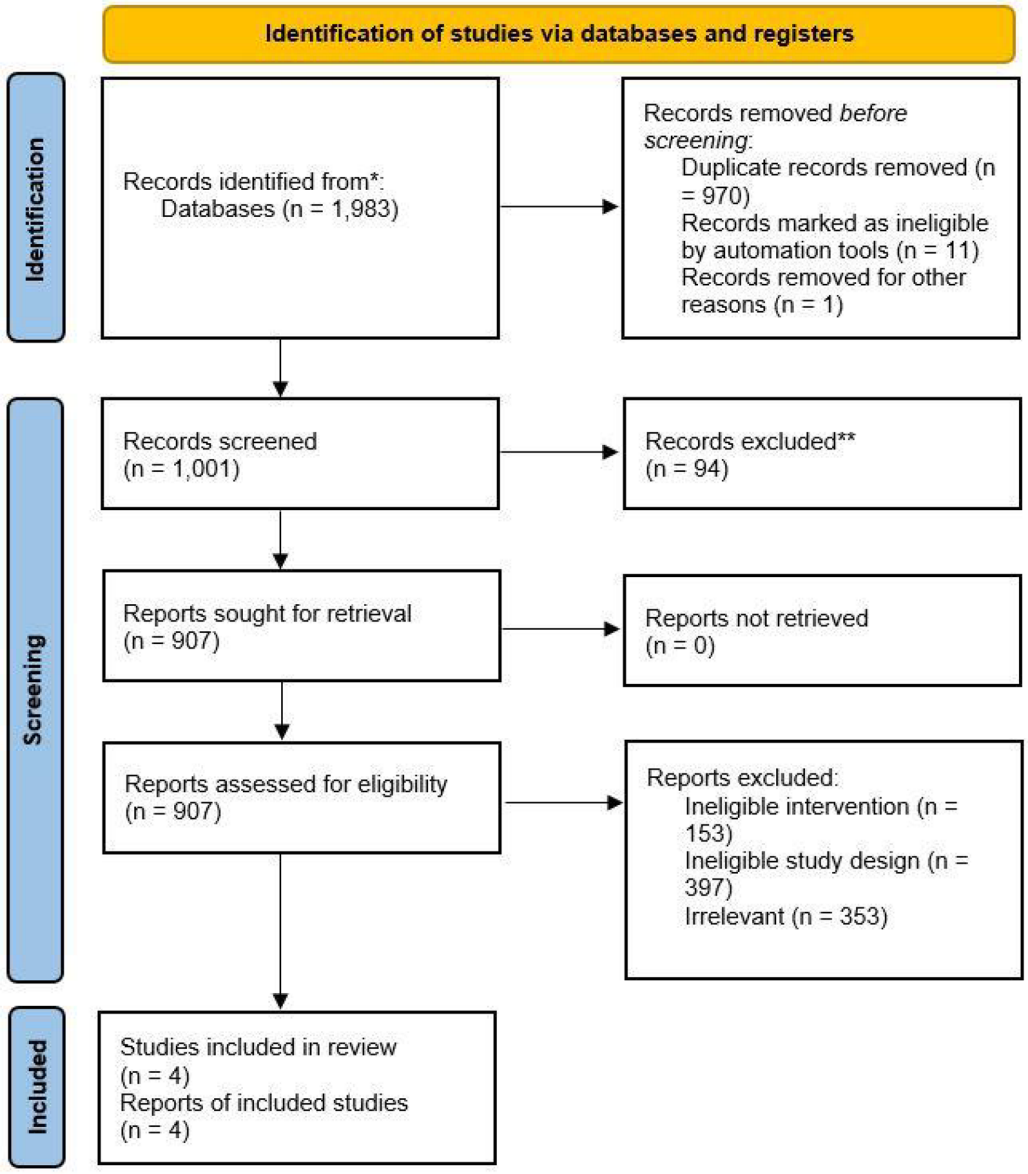
PRISMA flowchart

The initial literature search yielded 1983 studies which after the removal of duplicates (*n* = 970) and eligible studies, yielded 907 articles for the full-text review. Finally, four randomized controlled trails (RCTs) were included in the final qualitative and quantitative analysis. Further details are available in the PRISMA flowchart (Fig. 1). Empagliflozin, dapagliflozin, and sotagliflozin were the SGLT2 inhibitors tested in the included RCTs. The total number of patients were 14,676, with 40.2% (*n* = 5,900) of the patient population being women. Of the included patients, 55.6% and 52.8% had diabetes and renal impairment at baseline, respectively. Further details are available in Table 1.

**Table 1:** Baseline patients’ characteristics table

		EMPEROR-reduced		DAPA-HF		EMPEROR-preserved		SOLOIST-WHF	
		Empa	Pla	Dapa	Pla	Empa	Pla	Sota	Pla
*N*		1863	1867	2373	2371	2997	2991	608	614
Age, years (SD)		67.2 (10.8)	66.5 (11.2)	66.2 (11.0)	66.5 (10.8)	71 (9)	72 (10)	198 (33)	214 (345)
Women, *n* (%)		437 (23.5)	456 (24.4)	564 (23.8)	545 (23.0)	1338 (44.6)	1338 (44.7)	608 (100)	614 (100)
HF, n (%)	Overall	1863 (100)	1867 (100)	2373 (100)	2371 (100)	2997 (100)	2991 (100)	481 (79)	485 (79)
	HFrEF	1863 (100)	1867 (100)	2373 (100)	2371 (100)	–	–	127 (21)	129 (21)
	HFpEF	–	–	–	–	2997 (100)	2991 (100)	127 (21)	129 (21)
Diabetes, *n* (%) ‡		927 (49.8)	929 (49.8)	1075 (45.3)	1064 (44.9)	1466 (48.9)	1472 (49.2)	608 (100)	614 (100)
eGFR, mL/min per 1.73m2§, mean (SD)		61.8 (21.7)	62.2 (21.5)	66.0 (19.6)	65.5 (19.3)	60.6 (19.8)	60.6 (19.9)	49	51
Renal Impairment		981 (52.6)	997 (53.4)	962 (40.5)	964 (40.7)	1504 (50.2)	1484 (49.6)	854 (70)	
NT-proBNP, pg/mL		1887 (1077–3429)	1926 (1153–3525)	1428 (857–2655)	1446 (857–2641)	–	–	1817 (845–3659)	1741 (843–3582)
ACE inhibitor		867 (46.5)	836 (44.8)	1332 (56.1)	1329 (56.1)	2428 (81.0)	2404 (80.4)	254 (41.8)	241 (39.3)
ARB		451 (24.2)	457 (24.5)	675 (28.4)	632 (26.7)	–	–	245 (40.3)	270 (44.0)
Mineralocorticoid receptor antagonist		1306 (70.1)	1355 (72.6)	1696 (71.5)	1674 (70.6)	1119 (37.3)	1125 (37.6)	403 (66.3)	385 (62.7)
Diuretic		482	3174	2216 (93.4)	2217 (93.5)	–	–	646	643
B-blocker		1765 (94.7)	1768 (94.7)	2278 (96.0)	2280 (96.2)	2598 (86.7)	2569 (85.9)	564 (92.8)	561 (91.4)
Statin		2554	1176	3176	1568	2042 (68.1)	2089 (69.8)	-	-
ARNI		340 (18.3)	387 (20.7)	250 (10.5)	258 (10.9)	65 (2.2)	69 (2.3)	93 (15.3)	112 (18.2)

*HF* heart failure, *HFrEF* heart failure with reduced ejection fraction, *HFpEF* heart failure with preserved ejection fraction, *eGFR* estimated glomerular filtration rate, *NT-proBNP N*-terminal pro-natriuretic peptide, *ACE inhibitors* angiotensin-converting enzyme inhibitors, *ARB* angiotensin receptor blocker

### Efficacy

#### Composite of cardiovascular death or hospitalization due to heart failure

**Fig. 2:**
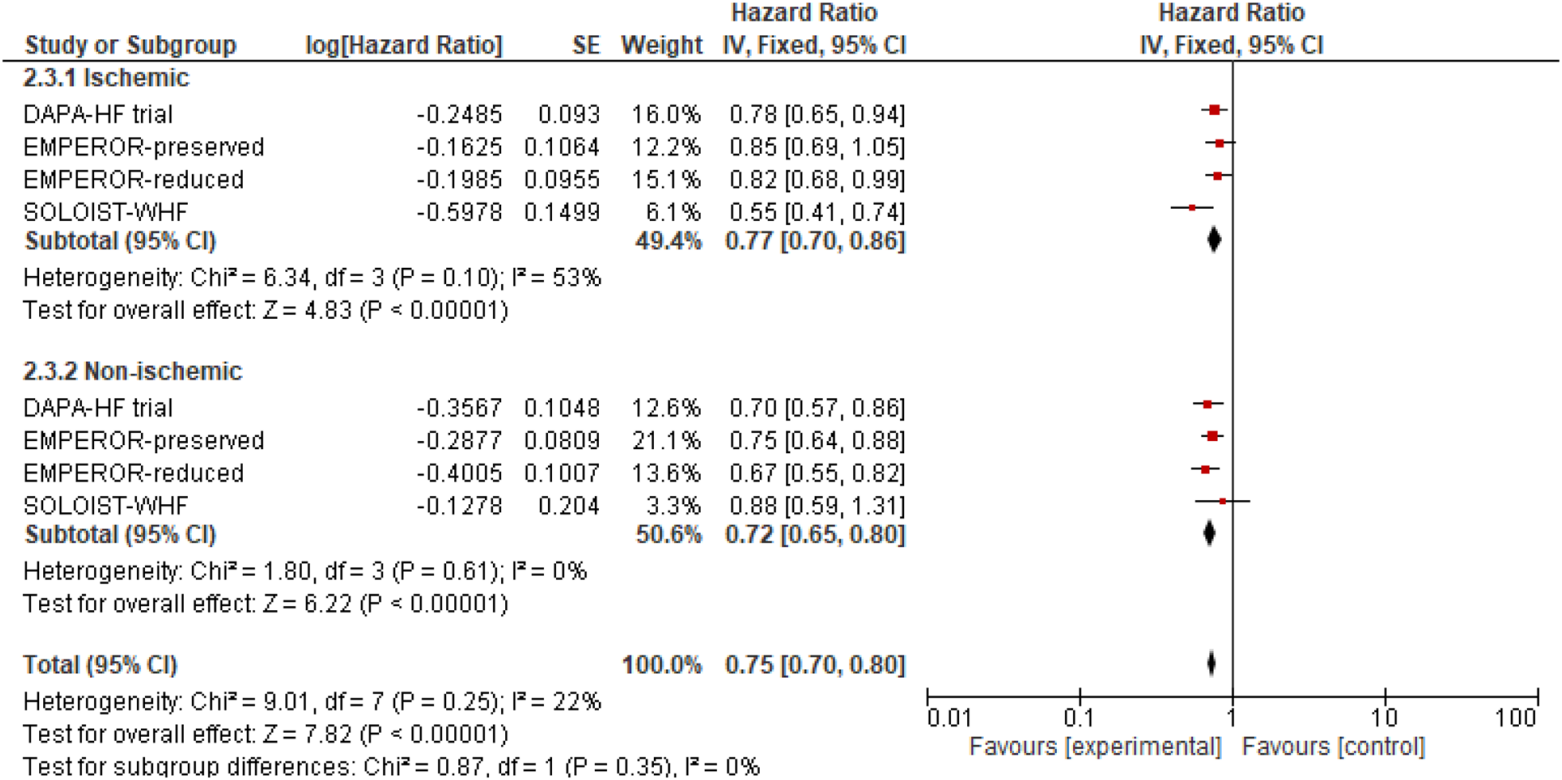
Cardiovascular death (CVD) or hospitalization due to heart failure (HHF)

This outcome was reported by four RCTs. When compared to the placebo group, SGLT2i showed significant reduction in incidences of CVD or HHF in both the ischemic group (HR: 0.77 (95% CI 0.70, 0.86) *P* < 0.00001, *I*^2^ = 53%) and the non-ischemic group (HR: 0.72 (95% CI 0.65, 0.80) *P* < 0.00001, *I*^2^ = 0%). Although there was greater risk reduction in the non-ischemic group (23%), compared to the ischemic group (28%), no statistically significant difference was observed between the two subgroups (*P*-interaction = 0.35) (Fig. 2).

#### Hospitalization due to heart failure

**Fig. 3:**
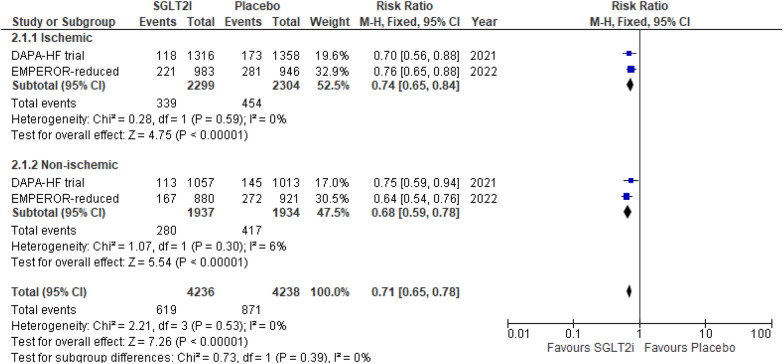
Hospitalization due to heart failure (HHF)

Only two RCTs reported this outcome. SGLT2i showed a 26% reduction in the risk of HHF in the ischemic group (RR 0.74 (95% CI 0.65, 0.84) *P* < 0.00001, *I*^2^ = 0%) and a 32% reduction in risk of HHF in the non-ischemic group (RR 0.68 (95% CI 0.59, 0.78) *P* < 0.00001, *I*^2^ = 6%). Although patients in the non-ischemic group showed greater improvement in this outcome as compared to ischemic group, the differences in the subgroup finding remain nonsignificant (*P*-interaction = 0.39) (Fig. 3).

#### Cardiovascular death

**Fig. 4:**
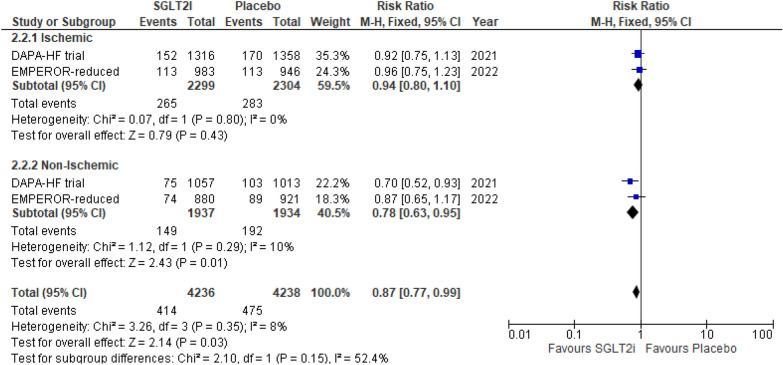
Cardiovascular death (CVD)

This outcome was reported by only two RCTs. Although SGLT2i showed a significant reduction in incidence of CVD in the non-ischemic group (RR 0.78 (95% CI 0.63, 0.95) *P* = 0.01, *I*^2^ = 10%), the results were nonsignificant for the ischemic group (RR 0.94 (95% CI 0.80, 1.10) *P* = 0.43, *I*^2^ = 0%). Despite superior outcomes in the non-ischemic group, the subgroup differences between both the HF etiologies remained insignificant (*P*-interaction = 0.15) (Fig. 4).

#### All-cause mortality

**Fig. 5:**
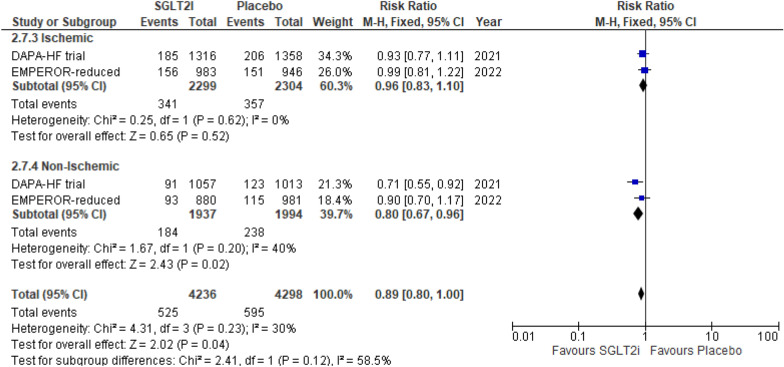
All-cause mortality

Two RCTs reported this outcome. As compared to placebo, SGLT2i showed a significant reduction in incidence of all-cause mortality in the non-ischemic group (RR 0.80 (95% CI 0.67, 0.96) *P* = 0.02, *I*^2^ = 40%); however, the change was nonsignificant for the ischemic group (RR 0.96 (95% CI 0.83, 1.10) *P* = 0.52, *I*^2^ = 0%). Despite superior outcomes in the non-ischemic group, the subgroup differences between both the HF etiologies remained insignificant (*P*-interaction = 0.15) (Fig. 5).

### Safety

#### Volume depletion

**Fig. 6:**
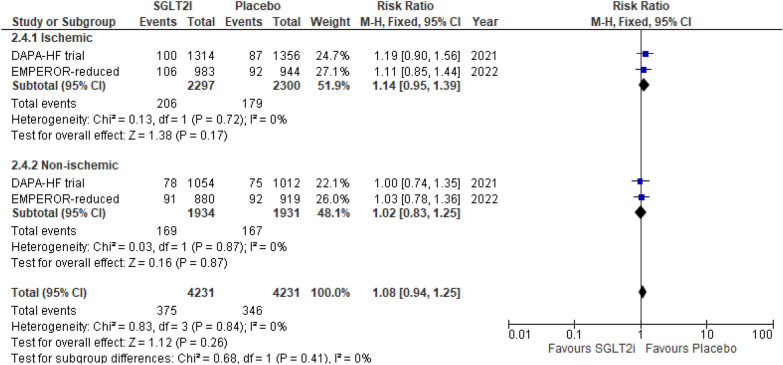
Volume depletion

Reported by two RCTs, there was an insignificant difference between SGLT2i and placebo reporting this outcome in both the ischemic group (RR 1.14 (95% CI 0.95, 1.39) *P* = 0.17, *I*^2^ = 0%) and non-ischemic group (RR 1.02 (95% CI 0.83, 1.25) *P* = 0.87, *I*^2^ = 0%) (*P*-interaction = 0.41) (Fig. 6).

#### Fracture

**Fig. 7:**
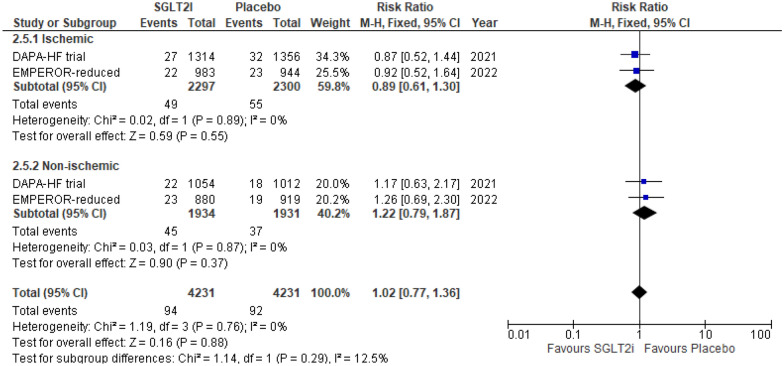
Fracture

This outcome was reported by two RCTs. There was an insignificant difference between SGLT2i and placebo reporting fracture in both the ischemic group (RR 0.89 (95% CI 0.61, 1.30) *P* = 0.55, *I*^2^ = 0%) and non-ischemic group (RR 1.22 (95% CI 0.79, 1.87) *P* = 0.37, *I*^2^ = 0%) (*P*-interaction = 0.29) (Fig. 7).

#### Discontinuation of drug due to adverse events

**Fig. 8:**
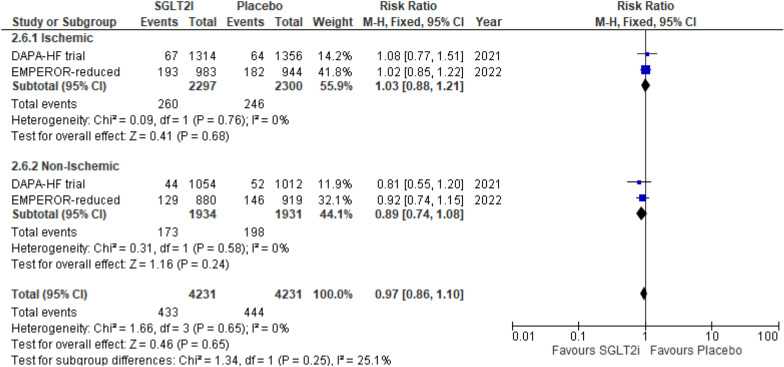
Discontinuation of drug due to adverse events

Only two studies reported this outcome. SGLT2i when compared to placebo showed no significant difference in this discontinuation of drug due to adverse events in both, the ischemic group (RR 1.03 (95% CI 0.88, 1.21) *P* = 0.68, *I*^2^ = 0%) and non-ischemic group (RR 0.89 (95% CI 0.74, 1.08) *P* = 0.24, *I*^2^ = 0%) (*P*-interaction = 0.25) (Fig. 8).

### Publication bias and quality assessment

**Fig. 9:**
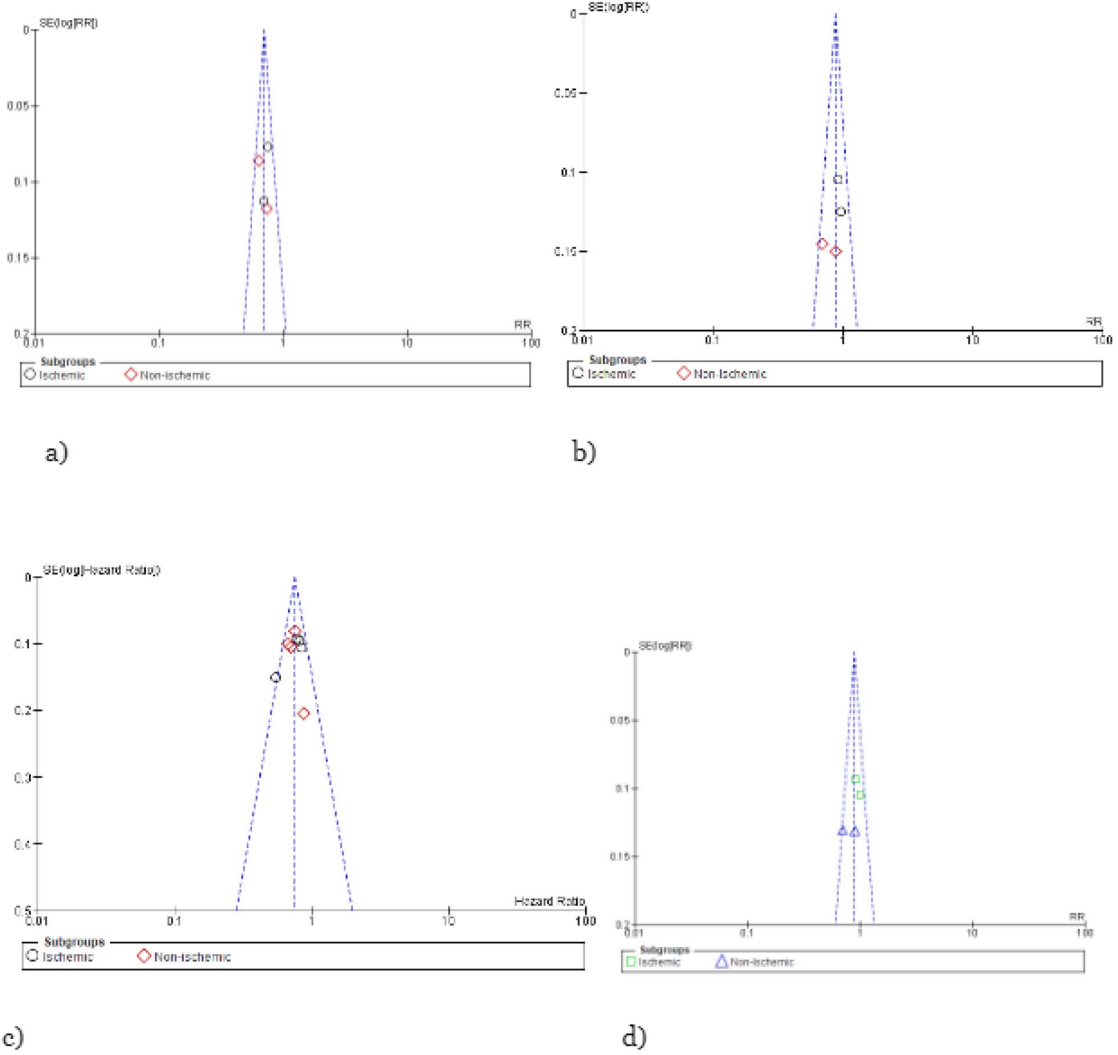
Forest plots for the following outcome: **a** HHF, **b** CVD, **c** CVD/HHF, and **d** all-cause mortality

**Fig. 10:**
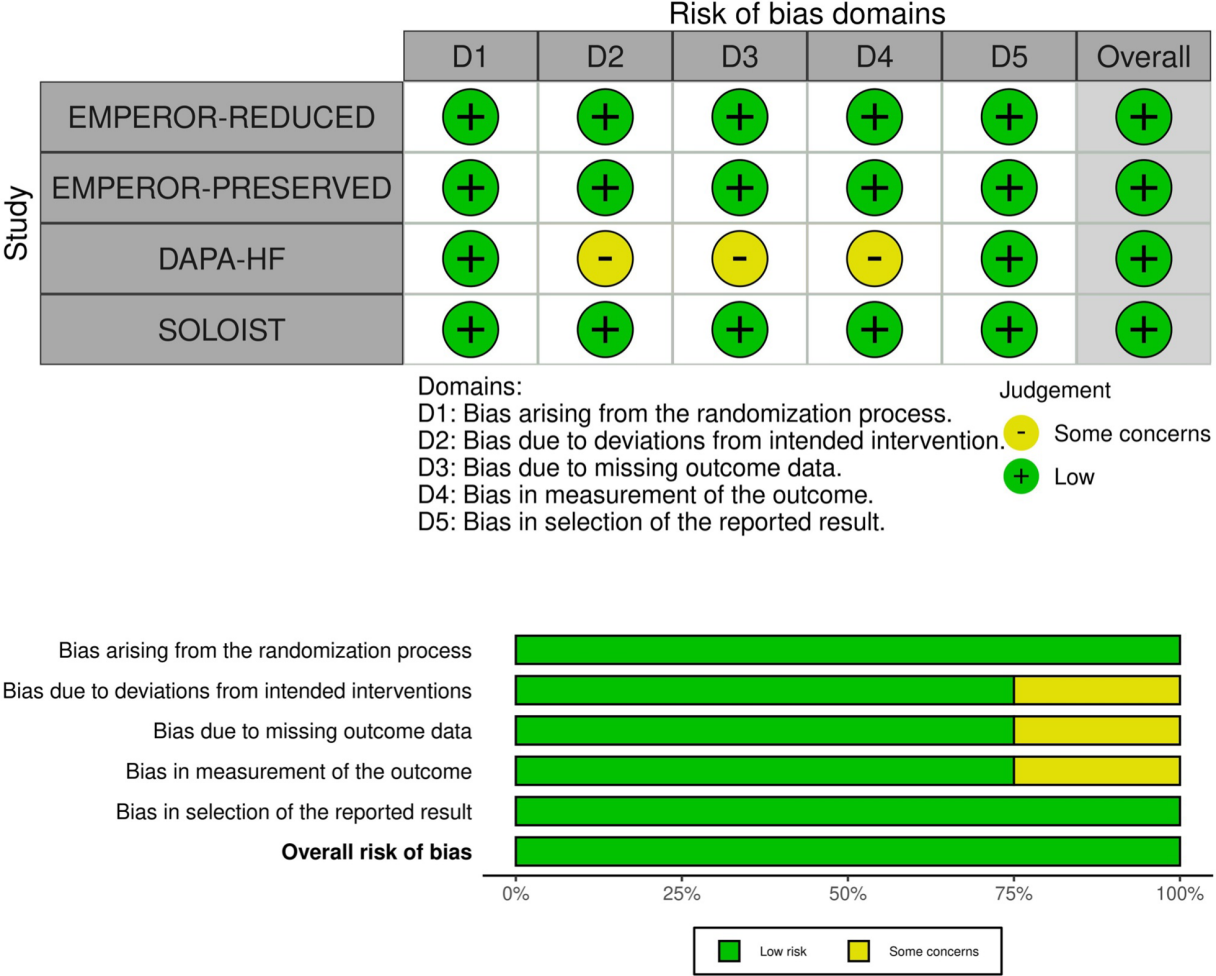
Cochrane RoB 2 traffic light and summary plots

Visual inspection of funnels plots did not reveal and potential publication bias (Fig. 9). All the RCTs showed a low overall risk of bias in Cochrane’s ROB assessment. Further details are available in Fig. 10.

## Discussion

**Fig. 11:**
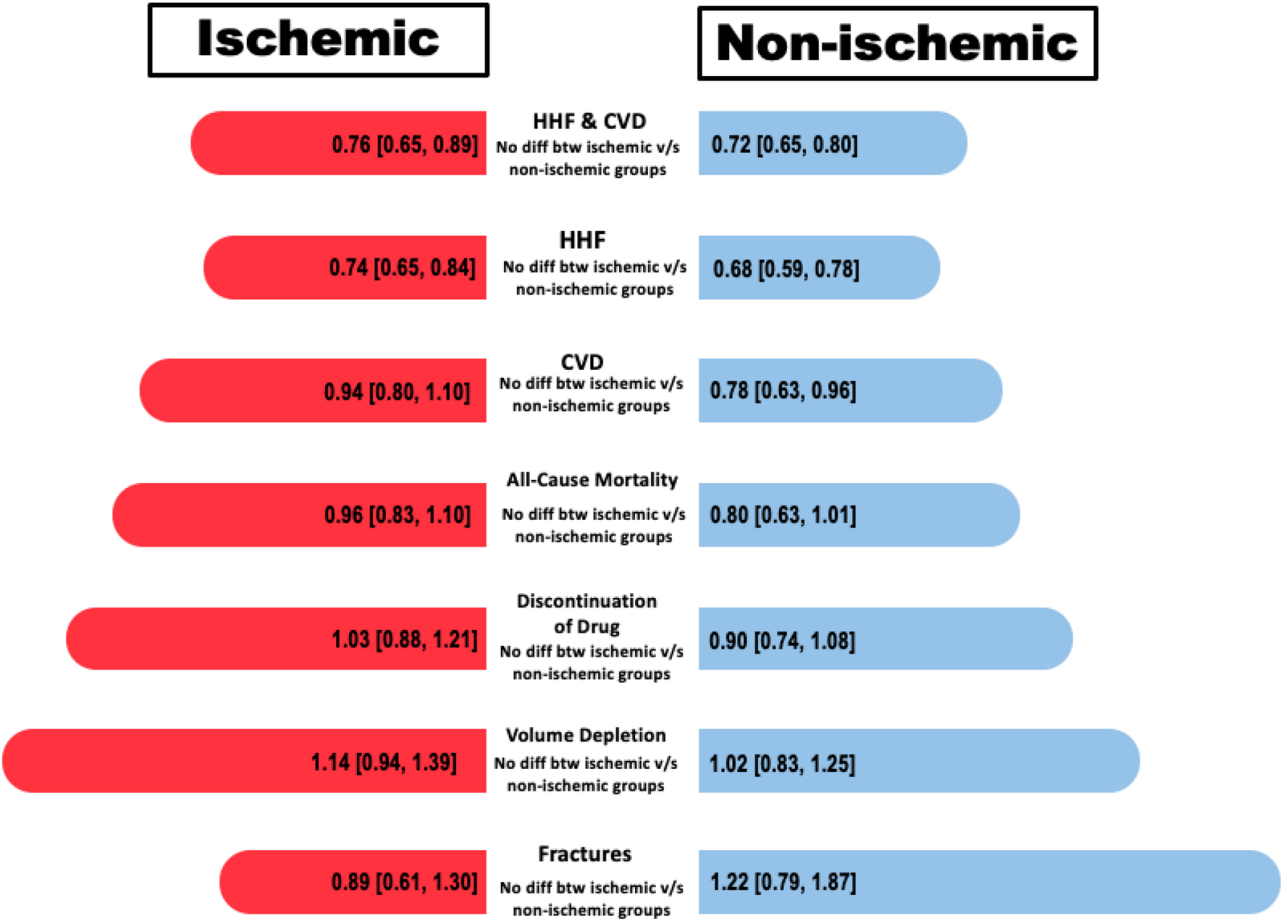
Graphical illustration

The aim of this systematic review and meta-analyses was to assess the safety and efficacy of SGLT2i in HF patients stratified according to etiology of HF (ischemic vs. non-ischemic). The summary of our findings encompassing a substantial cohort of over 14,000 patients has unveiled several noteworthy findings (Fig. 11). Firstly, both the ischemic and non-ischemic patient groups exhibited a statistically significant reduction in CVD and HHF when compared to the placebo group. Secondly, when analyzing outcomes such as CVD and all-cause mortality, the non-ischemic group displayed a significant decrease, whereas the ischemic group did not show a statistically significant effect, despite an overall improvement in both CVD and all-cause mortality. Lastly, in terms of undesirable safety events, including fractures, volume depletion, and discontinuation of the drug due to adverse events, patients in the SGLT2i group did not exhibit a significant difference when compared to the placebo group. However, it is important to note that, for all outcomes except CVD/HHF, the limited number of studies reporting these outcomes may potentially reflect inadequate statistical power rather than a genuine lack of effect.

In our analysis of four RCTs, SGLT2 inhibitors significantly reduced the combined occurrence of CVD/HHF, with a more pronounced effect in the non-ischemic group. Importantly, despite the absence of statistical significance between the groups, our findings endorse the use of SGLT2 inhibitors in HF patients, regardless of etiology, in alignment with current literature. Multiple studies, including one by J. Butler et al., consistently reported a substantial reduction in the composite outcome of first HHF/CVD events with SGLT2 inhibitor use. J. Butler et al. reported an HR of 0.72, highlighting a significant decrease in the risk of such events [14]. These results are corroborated by a systematic review conducted by S. Raza and colleagues, reinforcing the consistent reduction in cardiovascular endpoints, including CVD and HHF [15]. However, a persistent puzzle remains regarding why no discernible difference in treatment outcomes has been established, despite evidence that HF attributed to ischemic causes typically carries a bleaker prognosis than non-ischemic origins [16, 17]. Furthermore, ischemic etiology serves as a robust predictor for the composite endpoint of CVD/HHF, irrespective of gender [17]. One plausible explanation for this inconsistency could be attributed to the older age and a higher burden of comorbidities often observed in patients with ischemic HFrEF. These factors may significantly influence prognosis and have raised concerns about the effectiveness of therapeutic interventions. While it is possible that sicker patients with a higher baseline risk may exhibit a greater response to specific treatments, those with scarred and nonviable myocardium may be less amenable to improvement [8]. The variability in ischemic etiology may thus contribute to the diverse treatment responses observed.

Additionally, our analysis concluded that SGLT2 inhibitors reduced CVD and all-cause mortality significantly in non-ischemic HF but not in ischemic HF, with no significant subgroup differences between the two etiologies. Despite evidence supporting the narrative that individuals with ischemic HFrEF exhibit an increased risk of CVD and all-cause mortality after adjusting for significant prognostic factors [17, 18], the current literature challenges this perspective based on recent trials like the DAPA-HF (Dapagliflozin and Prevention of Adverse Outcomes in Heart Failure; NCT03036124) and the EMPEROR-Reduced (Empagliflozin Outcome Trial In Patients With Chronic Heart Failure With Reduced Ejection Fraction; NCT03057977), which have demonstrated similar all-cause and cardiovascular benefits among HF patients with either etiology [19–21]. Several factors contribute to the ambiguity surrounding this issue. Firstly, the original classification of HF etiology is inherently subjective and lacks a widely agreed-upon definition. Secondly, the presence of various comorbidities further complicates both the etiology and expected treatment outcomes.

Despite conflicting reports on HF prognosis based on etiology, our findings are in conformity with multiple recent trials on HF. The EMPHASIS-HF (Eplerenone in Mild Patients Hospitalization and Survival Study in Heart Failure) trial, the MERIT‐HF (Metoprolol CR/XL Randomized Intervention Trial in Congestive Heart Failure) trial, and the PARADIGM‐HF (Prospective Comparison of Angiotensin Receptor-Neprilysin Inhibitor With Angiotensin‐Converting Enzyme Inhibitor to Determine Impact on Global Mortality and Morbidity in Heart Failure) trial all manifested similar cardiovascular outcomes in HF patients, with no significant differences between the ischemic and non-ischemic subgroups [22–24]. Our analysis sheds light on the use of SGLT2 inhibitors in HF, revealing their consistent effectiveness across different etiologies. This parallels the experience with other pharmacotherapies for HFrEF, where efficacy remains stable regardless of the underlying causes.

Looking forward, it is worth contemplating the potential implications of etiology in the context of cardiac devices. Studies have suggested that prophylactic implantation of cardioverter-defibrillators can reduce mortality rates in individuals with ischemic HF and reduced ejection fraction [8]. This underscores the possibility of tailoring interventions based on etiological factors to further enhance patient outcomes.

To ensure more consistent and well-founded outcome's assessment, a detailed analysis of remaining SGLT2i trials and new trials stratifying patients based on their baseline HF etiology are necessary a prerequisite. Since the patient population in this study was predominantly those with HF, newer studies focused on a combination of HF, type 2 diabetes (T2DM), and chronic kidney disease (CKD) at baseline will allow for a more holistic approach to treat patients presenting with a variation of interconnected comorbidities. Furthermore, as indicated by Adam et al., understanding the gender-by-etiology interaction in HF patients is vital. While HF etiology remains the strongest predictor of survival, gender's role in prognostic accuracy requires further exploratory research [25]. These endeavors can shape the future of HF management by offering tailored therapies and insights into gender-specific outcomes, ultimately improving patient care.

### Study limitations

First, the number of included RCTs was limited to four, which might affect the overall statistical power and generalizability of our findings. Additionally, while we aimed to analyze the effects of SGLT2i on HF patients stratified by ischemic and non-ischemic etiologies, the available data were derived from a subset of trials that reported these specific stratifications, potentially introducing selection bias. Moreover, HF etiology based outcomes were investigator reported so there is a chance of misclassification of cause of HF due to lack of strict instructions [26]. Despite these limitations, our meta-analyses provides valuable insights into the safety and efficacy of SGLT2i in HF patients, emphasizing the need for further research to address these limitations and enhance our understanding of treatment effects in different HF etiologies.

## Conclusion

Our analysis showed that SGLT2i is effective in reducing all-cause mortality, cardiovascular mortality, and associated adverse events in both ischemic and non-ischemic etiology of HF. However, considering the limited number of data available from few trails, more studies are requisite to warrant any clinically relevant decision-making.

## Data Availability

Not applicable.

## Abbreviations

SGLT2i: Sodium-glucose cotransporter 2 inhibitors
HF: Heart failure
HFH: Hospitalization due to heart failure
CVD: Cardiovascular death
RCTs: Randomized control trials
RR: Risk ratio
HR: Hazard ratio
ESC: European Society of Cardiology
HFrEF: Heart failure with reduced ejection fraction
PRISMA: Preferred Reporting Items for Systematic Reviews and Meta-Analyses
ROB: Risk of bias
